# Identification of in vivo induced maternal haploids in maize using seedling traits

**DOI:** 10.1007/s10681-017-1968-3

**Published:** 2017-07-14

**Authors:** Vijay Chaikam, Luis Antonio Lopez, Leocadio Martinez, Juan Burgueño, Prasanna M. Boddupalli

**Affiliations:** 1 International Maize and Wheat Improvement Center (CIMMYT), ICRAF campus, UN Avenue, Gigiri, P. O. Box 1041–00621, Nairobi, Kenya; 2 International Maize and Wheat Improvement Center (CIMMYT), Apdo. Postal 6-641, 06600 Mexico City, DF, Mexico

**Keywords:** Doubled haploid, In vivo induction, Maternal haploids, Seedling traits

## Abstract

In vivo haploid induction in high frequency followed by efficient identification of haploids are important components of deriving completely homozygous doubled haploid (DH) lines in maize. Several genetic marker systems were proposed and/or used for identification of in vivo maternal haploids in maize, such as *R1-nj* (Navajo), high oil, red root and transgenic markers. In this study, we propose a new method of haploid/diploid identification based on natural differences in seedling traits of haploids and diploids, which can be used in any induction cross independently of the genetic marker systems. Using confirmed haploids and diploids from five different populations, the study established that haploid and diploid seedlings exhibit significant differences for seedling traits, particularly radicle length (RL), coleoptile length (CL), and number of lateral seminal roots (NLSR). In six populations that exhibited complete inhibition of the commonly used *R1-nj* (Navajo) marker, we could effectively differentiate haploids from diploids by visual inspection of the seedling traits. In the haploid seed fraction identified based on *R1-nj* marker in ten populations, false positives were reduced several-fold by early identification of haploids at seedling stage using the seedling traits. We propose that seedling traits may be integrated at the haploid identification stage, especially in populations that are not amenable to use of genetic markers, and for improving the efficiency of DH line production by reducing the false positives.

## Introduction

Doubled haploid (DH) technology has become an invaluable tool in maize breeding programs to produce homozygous lines from heterozygous populations within just two cycles compared to 6–8 cycles through conventional inbreeding. Through production of DH lines, entire genetic variance present in the segregating populations can be accessed readily in completely homozygous condition from the very beginning of selection process (Melchinger et al. 2013). Use of DH lines offers several quantitative-genetic, economic and logistical advantages over conventional inbred lines and hence is being preferred in breeding programs and genetic studies (Röber et al. 2005; Prasanna 2012). Production of DH lines in maize involves in vivo induction of haploids, identification of haploids at seed or seedling stage, chromosomal doubling in haploid seedlings and production of seed for DH lines from fertile doubled haploid plants (Prasanna et al. 2012; Prigge and Melchinger 2012). One of the critical requirements for large scale production of DH lines in maize is efficient and accurate identification of a small proportion of haploids from the diploids among the seeds obtained through induction crosses, before subjecting the selected haploids to chromosomal doubling treatment (Chaikam et al. 2016). All of the currently used maternal haploid inducers in maize are equipped with an anthocyanin marker for haploid identification conditioned by R1-nj (Navajo) gene (Melchinger et al. 2015). When such R1-nj based inducers are crossed as male parent with the source germplasm/population (from which DH lines are desired) as the female parent, the resultant seeds will predominantly have diploids marked with anthocyanin coloration on both endosperm and embryo whereas the maternal haploids have anthocyanin coloration only on the endosperm (Nanda and Chase 1966; Greenblatt and Bock 1967). Even though the Navajo marker facilitates easy visual separation of haploids and diploids, it can be completely or partially inhibited in induction crosses by an anthocyanin inhibitor gene *C1-I* (Röber et al. 2005; Chaikam et al. 2015). In addition, Navajo based haploid versus diploid classification can result in high false positives (Röber et al. 2005; Prigge et al. 2011; Melchinger et al. 2014; Chaikam et al. 2016) and false negatives (Röber et al. 2005; Chaikam et al. 2016), and is not useful when the source germplasm has either color inhibitor *C1-I* or natural anthocyanin coloration (purple/red) on the pericarp/endosperm as is the case in many maize landraces (Chaikam et al. 2016).

Alternative marker systems proposed to identify in vivo induced maize haploids at seed stage include high oil (Rotarenco et al. 2007; Melchinger et al. 2013) or at an early seedling stage include red root (Rotarenco et al. 2010; Chaikam et al. 2016), transgenic herbicide resistance (Geiger et al. 1994), or transgenic green fluorescent protein marker (Yu and Birchler 2016). Similar to Navajo marker, all these marker systems need to be integrated into the haploid inducer lines in order to employ them for haploid induction and identification. Among these, transgenic marker systems may not be a practical solution in many countries because of the regulatory approval process and high cost of development. Advantages of the high-oil marker include early identification of haploids at the seed stage, genotype-independence and the possibility to automate haploid/diploid seed classification (Melchinger et al. 2013). Limitations are the need to develop/obtain inducers with high-oil trait, possibility of false positives and false negatives, and the initial cost of establishing an automated seed sorting system. Red root marker can effectively complement the Navajo marker in germplasm which inhibits anthocyanin coloration, as well as in germplasm that masks the Navajo marker expression (Chaikam et al. 2016). Limitations of the red root marker are (a) the necessity to germinate large number of seeds resulting from an induction cross, and (b) possibility of false positives and negatives. To achieve a relatively fool-proof haploid identification, it was recommended to integrate the high-oil and red root marker systems into inducer lines that are already equipped with Navajo marker, thereby enabling a pyramided-classification approach: first based on high-oil, second based on Navajo, and finally based on red root (Chaikam et al. 2016). Such an approach could potentially reduce the time and costs associated with haploid identification while significantly reducing the false positives. However, such haploid inducers, especially in the tropical genetic background, are not available as of now, whereas to develop/access an automation technology that enables use of high-oil marker could be costly. Considering these limitations with integration of phenotypic marker systems into haploid inducer lines, it would be of interest to identify traits that can aid in haploid identification independent of marker systems integrated in haploid inducers.

It was recently shown that seed weight of haploids is significantly lower than that of the diploids (Smelser et al. 2015). However, it was noted that a haploid/diploid classification system based on seed weight would be less efficient than color markers/high-oil marker based haploid identification, and developing an automation technology is crucial for its adaptation (Smelser et al. 2015). The possibility of differentiating haploids and diploids based on seedling traits was not explored earlier. Therefore, the objectives of the present study were: (1) to study the differences between haploids and diploid seedlings in terms of radicle length (RL), coleoptile length (CL), and number of lateral seminal roots (NLSR) during early growth stages; (2) to validate the use of such traits for haploid identification in populations with complete inhibition of Navajo marker; and (3) to determine if seedling traits can be effectively used for early identification of false positives.

## Materials and methods

### Induction crosses

All the populations used in this study were lowland tropical or subtropical germplasm, sourced from different breeding programs of CIMMYT in Mexico. These populations were planted in a haploid induction nursery in the summer cycle of 2015 at CIMMYT’s Agua Fria experimental station (20.26°N, 97.38°W), as described in Chaikam et al. (2012). The source populations was crossed with bulked pollen from TAIL9 × TAIL8 hybrid inducer three consecutive days after silk emergence as described in Chaikam et al. (2012).

### Investigating the differences between haploid and diploid seedling traits

Seed produced from the induction crosses of five populations (population 1: CL106728/LH212Ht//CML451, population 2: ((CML491/LAPOSTASEQC7-F64-2-6-2-1-B-B)/CML491)-B-50-1-2-1-1-B/(CL G2312/CML9)-B-80-1-1-1-B, population 3: (CLG2312/CML505)-B-43-1-1-1-B/(CLRCW79/CLRCW98)-B-22-3-1-1-B-B, population 4: (LaPostaSeqC7F1021311BBBBCL04934)DH1 × CLWN247, and population 5: (LaPostaSeqC7F1021311BBBBCL04934)D H1 × CML551)) were used in this experiment as all these induction crosses showed excellent expression of Navajo marker (data not shown). Haploid and diploid seeds were separated based on Navajo phenotype, where the diploid seeds have purple coloration on the endosperm and embryo while the haploid seeds have purple coloration only on the endosperm (Chaikam and Prasanna 2012). Twenty seeds each were randomly chosen among the haploid and diploid fractions from each population and were germinated in paper towels for 72, 94 and 104 h in a growth chamber maintained at 28–30 °C, based on protocol described by Chaikam and Mahuku (2012). RL and CL were measured from the point of visible emergence to the tip using a meter scale. NLSR was counted at each time point. After measurements were done, the seedlings were transplanted in the field in rows spaced at 75 cm with a plant-to-plant spacing of 20 cm. After 30 days in the field, true ploidy status of each plant was confirmed for each surviving plant based on adult plant characteristics (plant vigor, erectness and paleness of leaves that can typically differentiate haploids from diploids). Haploids are generally weak with erect, narrow and pale leaves compared as to their diploid counterparts (Chase 1964; Prigge et al. 2011; Melchinger et al. 2013; Xu et al. 2013). Data collected on the confirmed haploids and the diploids was used for further analysis. A linear model was used to test the significance of differences between haploids versus diploids, as well as among different populations, and different time points, assuming normal distribution of the errors for RL and CL. The statistical model was:

$$y_{ijkl}=\mu +\alpha_{i}+\beta_{j}+\alpha \beta_{ij}+\delta_{k}+\alpha \delta_{ik}+\beta \delta_{ik}+\alpha \beta \delta_{ijk}+\epsilon_{ijkl}$$

where *Y_ijkl_* response variable, *l* overall mean, *α_i_* effect of the ith population, *β_j_* effect of the jth ploidy type, *δ_k_* effect of the kth hour, *αβ_ij_, βδ_jk_* and *αβδ_ijk_* are the interactions of the respective effects,*ε*~*N*(0,σ^2^) .

Least squares method was used to perform estimation and testing, F test was used for overall effect testing and t test to compare levels of the factors. Results are presented as mean squares, F values and significance values.

For the NLSR a generalized linear model with poisson distribution and log link was used. The used model was similar as the model described for RL and CL with the difference being that the response variable follows a poisson distribution and that the response variable is the log of NLSR. Maximum likelihood method was used to perform estimation and testing, as above; *F* and *t* tests were used to compare the overall effects and levels of the effects. Due to the method used, mean squares are not available, and only F values and related P values are shown.

### Use of seedling traits for haploid/diploid classification in induction crosses with inhibition of *R1-nj*

Induced seed from six populations that showed complete inhibition of Navajo phenotype were selected for this experiment. From each of the population, 1500 seeds were germinated in paper towels for 96 h, as described above. Seedlings were separated into haploids, diploids and undetermined classes based on visual assessment of seedling traits, primarily CL and RL. Additional traits that were used include NLSR, thickness of coleoptiles, thickness of radicles, and extent of root hairs. High proportion of germinated seedlings were vigorous with long coleoptiles, long radicles, and higher NLSR—traits typical of putative diploids. However, a smaller proportion of seedlings showed significantly lesser CL, RL and NLSR. Such seedlings also showed relatively thin coleoptiles, thin radicles and low number of root hairs. These were classified as putative haploids. Seedlings that cannot be easily classified into either putative haploids or putative diploids based on seedling traits were classified into an undetermined class. Separated putative haploids, putative diploids and undetermined seedlings were transplanted in the field to establish true ploidy status based on adult plant characteristics through the gold standard test based on plant vigor, erectness and paleness of the leaves, as described in Melchinger et al. (2013) and Chaikam et al. (2016). False discovery rate (FDR), false negative rate (FNR) and Matthews correlation coefficient (MCC) were also calculated (Melchinger et al. 2013; Chaikam et al. 2016). FDR is the proportion of diploids in the fraction classified as putative haploids; FNR is the proportion of true haploids misclassified as diploids; and MCC indicates the correlation between the real and predicted ploidy status based on the seedling traits.

### Use of seedling traits for identification of false positives resulting from *R1-nj* marker based haploid identification

To determine if false positives can be reduced by early detection of diploids in haploid fraction (identified based on Navajo marker), 500 putative haploid seeds each identified using Navajo marker expression were germinated from ten populations in paper towels, as described above. After 96 h of germination, seedlings were separated into haploids and diploids based on seedling traits as described above. Separated putative haploids and putative diploids were transplanted in the field and true ploidy status of each surviving plant was ascertained based on the gold standard test using adult plant characteristics, as described earlier.

## Results

### Differences between haploids and diploids

**Fig. 1:**
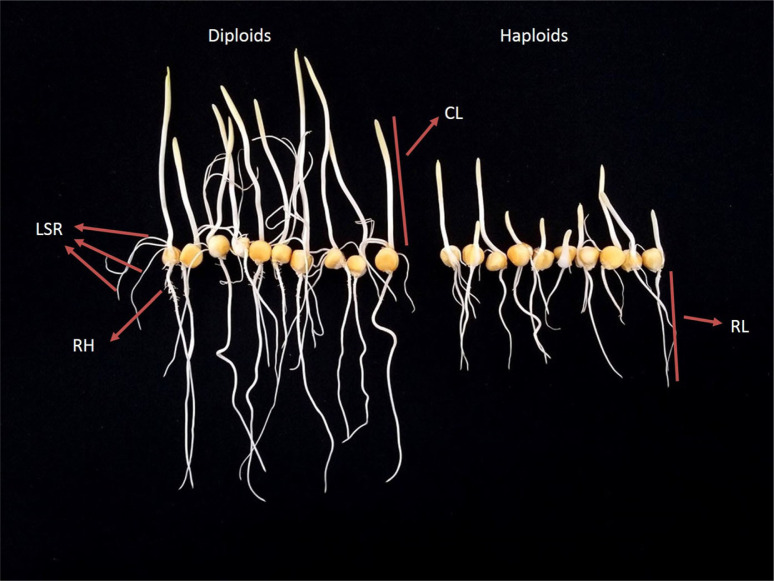
Morphological differences between diploid and haploid seedlings from population CL106728/LH212Ht//CML451 germinated for 96 h. The traits depicted include radicle length (RL), coleoptile length (CL), lateral seminal roots (LSR), and root hairs (RH)

Significant differences could be noted among the diploids and haploids for RL, CL, NLSR, thickness of coleoptile, thickness of radicle and presence of root hairs upon visual inspection (Fig. 1). In five populations studied, haploids tend to have shorter roots, shorter coleoptiles, and less NLSR compared to their diploid counterparts (Online Resource 1). As expected, CL and RL increased with increasing time of germination, while the NLSR remained same across time points (Online Resource 1). Statistical analysis (Table 1) revealed very significant effects (P > .0001) in RL, CL and NLSR for haploid/diploid classes of seedlings, however in the case of RL and CL the magnitude of the effect depended on the hour at which the measurements were taken but always with smaller values of RL and CL in haploid seeds. There were also highly significant differences between populations for RL (P > .001) and NLSR (P > .0001) and for CL (P > .0001) but in this case differences between populations depended on the hour at which the measurements were taken. No significant effects were found for the interactions of populations × ploidy of seedlings and for the interactions of genotype × class × time point for all the three traits studied.

**Table 1:** Statistical analysis of quantitative differences in RL, CL, and NLSR of confirmed haploid and diploid seedlings from five different populations

Source of variation	DF	Radicle length Mean square	Coleoptile length Mean square	Number of lateral seminal roots F value
Population (P)	4	32.2***	111****	6.36****
Ploidy (C)	1	4603****	1149****	33.9****
P × C	4	6.53NS	6.85NS	0.45NS
Hours of germination (H)	2	1263****	939****	1.72NS
P × H	8	10.1NS	5.99*	0.21NS
C × H	2	145****	89.1****	1.4NS
P × C × H	8	11.6NS	5.15NS	0.38NS
Error	522	6.2	1537	2.9

*DF* degrees of freedom, *NS* non-significant

* Significant at P B .05

** Significant at P B .01

*** Significant at P B .001

**** Significant at P B .0001

### Identification of haploids based on seedling traits in induction crosses with inhibition of *R1-nj*

Classification of haploids and diploids based on seedling traits in six populations with complete inhibition of Navajo marker resulted in FDR ranging from 9.2 to 39.4, at an overall average of 25.2. FNR ranged from 1.7 to 26.5, at an average of 12.7%. MCC values ranged from 0.64 to 0.94, at an average of 0.8. Overall, 11.8% of seedlings could not be easily classified into haploid and diploid classes. In such unclassified seedlings, haploid frequencies ranged from 12.7 to 50% at an average of 26.7% across all the populations (Table 2).

**Table 2:** Statistics associated with “gold standard” classification for differentiating putative haploids from putative diploids based on seedling traits

Source germplasm	N	FNR (%)	FDR (%)	MCC	Nu (%)	Haploids in Nu (%)
CML549/CLWN565	1295	1.7	9.2	0.94	11.1	29.2
CML549/CLWN630	1308	10.2	33.3	0.76	12.7	12.7
CLRCW96/CLWN630	1248	15.7	30.6	0.75	13.2	45.5
CLWQHZN58/CLWQHZN60	1893	26.5	39.4	0.64	9.88	34.2
CML491/CML150)/CML491)-B-21-1-1-1-1-B-B/(CLRCW79/CLRCW98)-B-22-3-1-1-B-B	2566	16.4	13.5	0.84	14.2	17.8
((LaPostaSeqC7F711212BBBBCL04934)DH10 9 CML551	1028	5.6	25.0	0.83	10.2	2.7
Overall	9338	12.7	25.2	0.8	11.8	23.7

*N* total number of plants assessed in “gold standard” test, *Nu* number of unclassified seedlings, *FNR* false negative rate, *FDR* false discovery rate, *MCC* Matthews correlation coefficient

### Use of seedling traits to identify false positives resulting from Navajo marker-based haploid versus diploid classification

When the Navajo marker is used for haploid/diploid classification in ten populations, FDR ranged from 9.3 to 43.0% at an average of 26.9% across all the populations. In the same populations, when the Navajo marker-based haploid identification was combined with identification of false positives using seedling traits, FDR values were considerably reduced in different populations ranging from 1.5 to 22.5% at an average of 9.4% across all populations. FDR decreased in the range of 1.7- to 6.3- fold with an average reduction by 3.5-fold when Navajo marker and seedling traits were combined, as compared to Navajo-based classification alone (Table 3).

**Table 3:** False discovery rates associated with Navajo marker-based haploid identification method versus combination of Navajo marker and seedling traits-based method of haploid identification

Source germplasm	N	*R1-nj* (%)	*R1-nj* + seedling traits (%)	Fold reduction
		FDR		
CLRCW96/CLWN564	409	34.0	7.0	4.8
CLWN425/CLWN564	422	31.5	6.9	4.6
CLWN425/CLWN630	330	18.1	6.1	3.0
CLWN425/CLWN603	313	24.3	8.7	2.8
CLWN564/CLWN603	328	19.5	11.4	1.7
POB 2 CML539-HIDAZO)	329	35.9	6.8	5.3
Fe&Zn 18n Diallele-2	249	43.0	22.5	1.9
Fe&Zn 19n Diallele-3	340	30.6	9.3	3.3
Fe&Zn 20n Diallele-4	263	22.8	13.8	1.7
Fe&Zn 21n Diallele-5	301	9.3	1.5	6.3
Overall	3284	26.9	9.4	3.5

*N* total number of plants assessed in gold standard test, *FDR* false discovery rate

## Discussion

It has been well-established that maternal haploid plants show reduced vigor, shorter stature, erect and narrow leaves, slender and weak stems, and reduced male fertility, as compared to their diploid counterparts (Chase 1964; Prigge et al. 2011; Melchinger et al. 2013; Xu et al. 2013). Differences in adult haploid and diploid plants are commonly used as a gold standard for ascertaining the ploidy status in several studies (Dong et al. 2013; Melchinger et al. 2013, 2015; Chaikam et al. 2016). However, for production of DH lines, it is very important to identify haploids either at the seed stage or at early seedling stage to reduce the cost involved in chromosomal doubling and management of large numbers of treated seedlings in the greenhouse and in the field. So, use of adult plant characters for haploid and diploid identification is not suited for large scale production of DH lines.

Our study clearly shows that young haploid seedlings can be effectively differentiated from the diploid seedlings through significant differences in RL, CL and NLSR compared to their diploid counterparts. As demonstrated with five populations, several seedling traits together can be used for distinguishing haploids from diploids among the seeds obtained through induction crosses. The FDR and FNR for such haploid/diploid classification were lesser than those reported for the commonly used Navajo marker system (Röber et al. 2005; Prigge et al. 2011; Melchinger et al. 2014; Chaikam et al. 2016); hence, the haploid/diploid identification is relatively more accurate using seedling traits as compared to using Navajo marker. However, a small proportion of seedlings (<10%) could not be easily classified into putative haploids or putative diploids using seedling traits, as they exhibited intermediate characteristics between haploids and diploids. Majority of such undetermined seedlings (on average 76.3%) turned out to be diploids through the gold standard test. Occurrence of diploid seedlings with intermediate characteristics and reduced vigor could be because of poor quality of some diploid seed or because of ear rot infection or insect damage, leading to slower germination and growth. The small proportion of haploids observed in this undetermined fraction (23.7% on average) of seedlings could be because of early spontaneous doubling in some haploids at very early stages, resulting in DH plants that are vigorous as compared to haploids; these plants exhibited relatively lesser vigor compared to diploids, as was also reported by Wu et al. (2014).

Even though seedling trait-based haploid/diploid classification can be more accurate, the method based on seedling traits requires germinating thousands of seedlings which could be labor- and time-intensive. For example, to obtain 1000 haploid seedlings one may need to germinate more than 10,000 seeds, assuming 10% haploid induction rate. Hence, it would be pragmatic to practice seedling trait-based haploid/diploid classification only in those populations that are not amenable for haploid identification using marker systems like Navajo or high oil or red root. Maize breeding programs without access to haploid inducers with alternative marker systems may benefit from using seedling traits for haploid identification.

Based on this study, we propose the following methodology for effectively distinguishing haploids from diploids using seedling traits for specific populations that are not amenable for Navajo-based marker system or for those breeding programs with lack of access to high-oil based marker system. First, putative diploid seedlings can be separated quickly based on visual assessment of most obvious traits of CL and RL. In our observation, seedlings that exhibit long coleoptiles always tended to have long radicles and turned out to be diploids in gold standard test. After separation of most obvious putative diploids, one can visually inspect the less vigorous and intermediate vigor seedlings for NLSR, extent of root hairs, width of radicle and width of coleoptile to separate haploids. A well-trained and experienced worker can visually inspect a seedling for different traits and classify it into different classes in less than 30 s. Hence, classification of haploids and diploids based on seedling traits takes similar time as classifying the seed based on Navajo marker. It is also possible to take photos of each paper bundle with germinating seedlings and employ image analysis tools to measure different seedling traits quickly and efficiently. A classification model based on measurements of different traits can be used to separate haploids and diploids which may increase accuracy and reduce FDR and FNR. However, efficient protocols for large-scale germination of induced seeds would enhance the efficiency of haploid/diploid classification based on seedling traits. Also, the proposed methodology cannot be used for haploid/diploid classification on a mixture of seedlings obtained from induction crosses of different populations as genotypes show significant differences for different seedling traits.

The more prominent application of this study would be identification of false positives resulting from use of any inducer with genetic marker systems. As demonstrated with ten populations in this study, use of seedling traits along with Navajo marker can reduce the FDR by several-fold compared to use of Navajo marker alone. Other methods proposed for identification of false positives include purple stem color marker (Röber et al. 2005), differences in guard cell measurements (Choe et al. 2012), flow cytometry (Dang et al. 2012; de Couto et al. 2013), and red root marker (Chaikam et al. 2016). Purple stem marker develops only after sufficient vegetative growth (Chaikam et al. 2016) and for conducting guard cell measurements and flow cytometry need sufficient growth to collect tissue. Hence, these methods cannot be practiced on early germinating seedlings and flow cytometry, guard cell measurement needs equipment like flow cytometers, microscopes etc., besides well-skilled personnel. Hence, these methods may not be of practical use in DH line production. For employing red root marker, haploid inducers equipped with this marker and adapted to specific environments are needed. Thus, seedling traits based identification of false positives could offer one of the practical options in identifying false positives at a very early stage before subjecting the seedlings to chromosomal doubling. This can improve the efficiency of DH line production by reducing the costs and labor involved in managing the false positives in greenhouse, treatment with costly chromosome-doubling chemicals and managing them in the field.

Together, seedling traits can be used as a standalone tool to identify in vivo induced maize haploids fairly accurately without needing any marker system integrated into the haploid inducer lines, and without requiring any expensive equipment and high technical expertise. Until efficient haploid inducers equipped with several phenotypic markers are developed and deployed in diverse agro-ecologies, this simple method of haploid identification can be effectively used to increase the efficiency of DH line development.

## Abbreviations

DH: Doubled haploid
FDR: False discovery rate
FNR: False negative rate
MCC: Matthews correlation coefficient
CL: Coleoptile length
RL: Radicle length
NLSR: Number of lateral seminal roots
RH: Root hairs

## Notes

**Open Access** This article is distributed under the terms of the Creative Commons Attribution 4.0 International License (http://creativecommons.org/licenses/by/4.0/), which permits unrestricted use, distribution, and reproduction in any medium, provided you give appropriate credit to the original author(s) and the source, provide a link to the Creative Commons license, and indicate if changes were made.
