# Supplementary figures and images for: Identification of in vivo induced maternal haploids in maize using seedling traits
Source: Euphytica. 2017 Jul 14;213:177. doi: 10.1007/s10681-017-1968-3 (PMC7734196; doi:10.1007/s10681-017-1968-3)

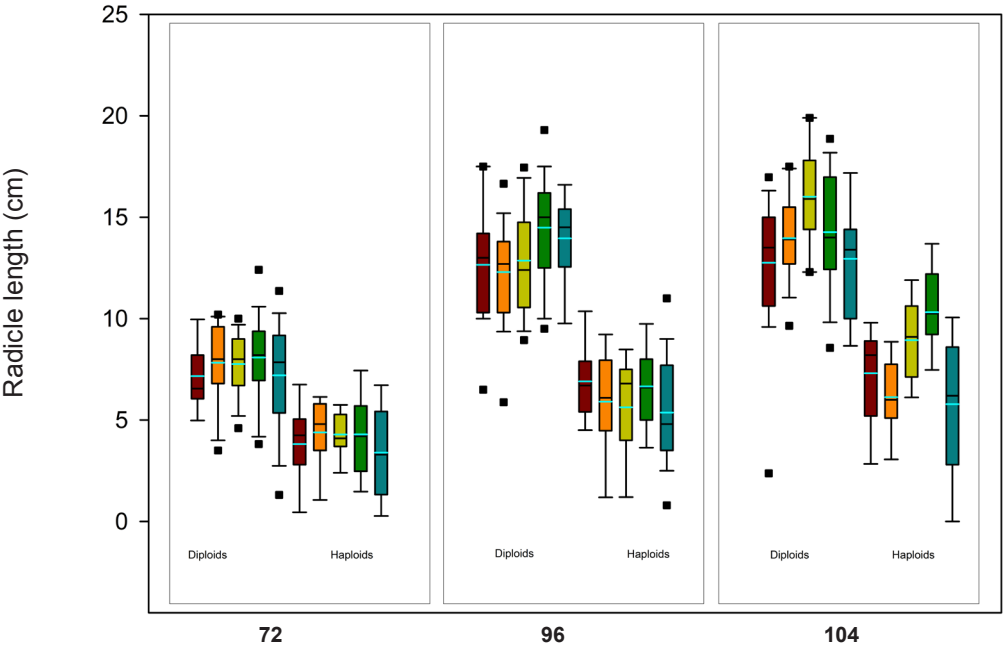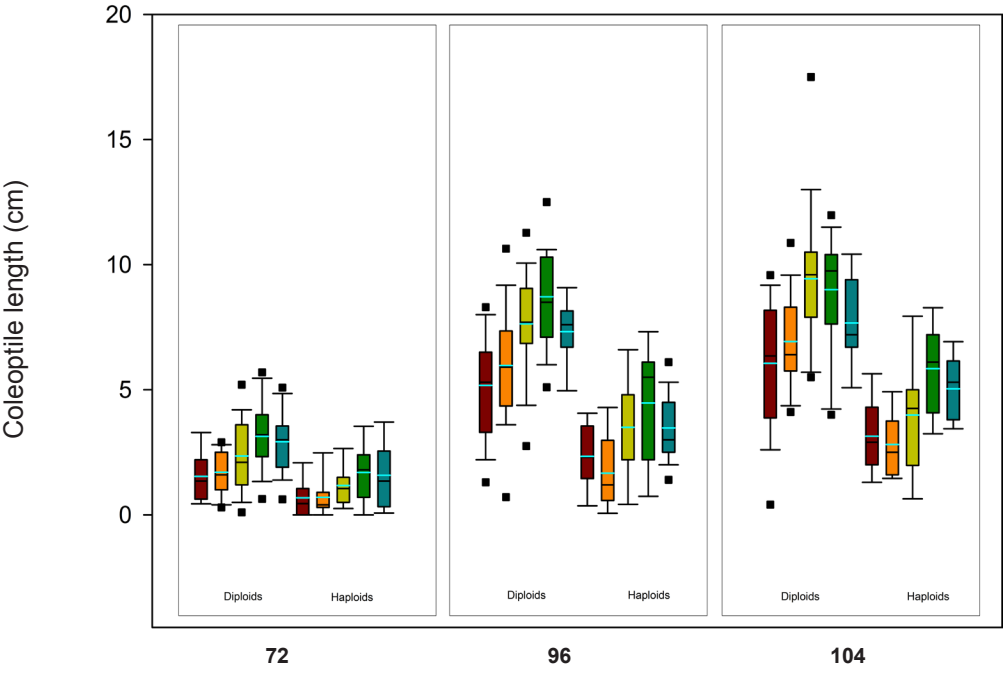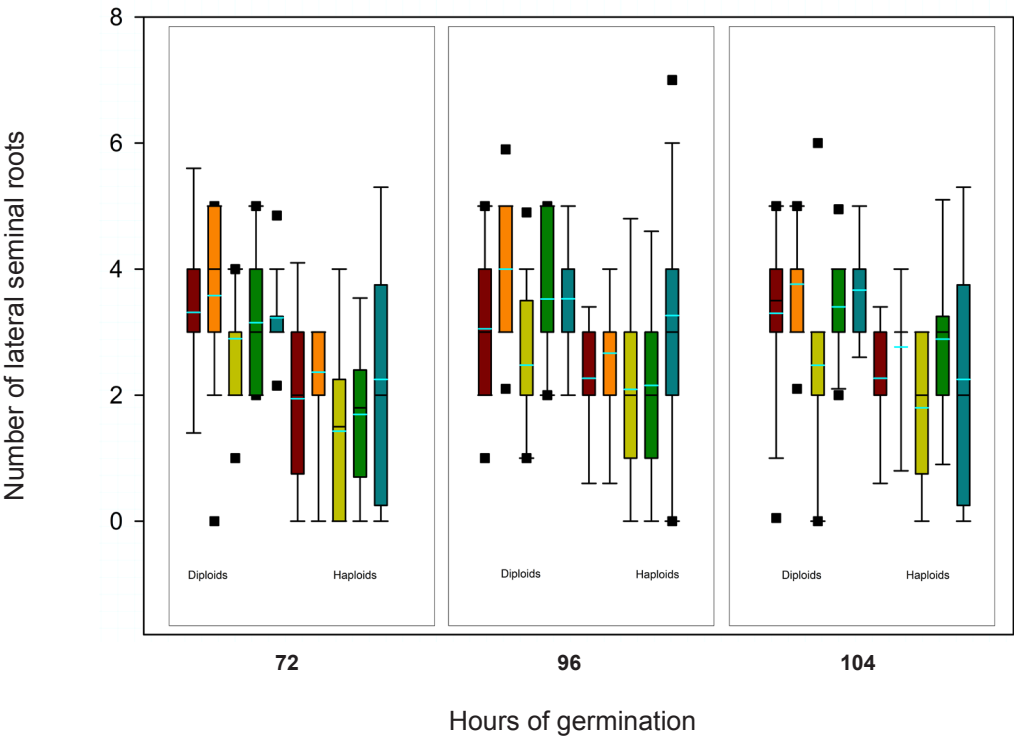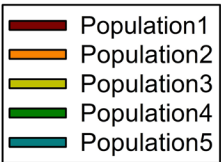

Supplement: Supplementary file 1 [file EUP-213-177-s001.pdf]
